# Short-Course Radiotherapy in Locally Advanced Rectal Cancer

**DOI:** 10.14309/ctg.0000000000000162

**Published:** 2020-06-08

**Authors:** Maria Cambray, Javier Gonzalez-Viguera, Miguel Angel Berenguer, Miquel Macià, Ferran Losa, Gemma Soler, Ricard Frago, J. Castellví, E. Guinó

**Affiliations:** 1 Catalan Institute of Oncology, Radiation Oncology Department, L'Hospitalet de Llobregat, Barcelona, Spain;; 2 Bellvitge University Hospital, General and Digestive Surgery Departement, L'Hospitalet de Llobregat, Barcelona, Spain;; 3 Moisès Broggi Hospital, General and Digestive Surgery Department, Sant Joan Despí, Barcelona, Spain;; 4 Catalan Institute of Oncology, Data Analytics Program, L'Hospitalet del Llobregat, Barcelona, Spain.

## Abstract

To date, we do not know the best therapeutic scheme in locally advanced rectal cancer when patients are older or have comorbidities.

**METHODS:**

In 2009, we established a prospective treatment protocol that included short-course preoperative radiotherapy (RT) with standard surgery +/− chemotherapy in frail patients, mostly older than 80 years or with comorbidities.

**RESULTS:**

We included 87 patients; the mean follow-up was 43.5 months (0.66–106.3). Disease-specific survival and disease-free survival at 36 months were 86.3% and 82.8%; at 60 months, they were 78.2% and 78%, respectively, with a local recurrence rate of 2.5%. The rate of late radiotoxicity was 9% in the form of sacral insufficiency fracture and small bowel obstruction with one death. The interval before surgery varied according to the involvement of the mesorectal fascia, but it was less than 2 weeks in 45% of cases. The rate of R0 was 95%. Surgical complications included abdominal wound dehiscence (3.5%), anastomotic leak (2.4%), and reoperations (11.5%). Downstaging was observed in 51% of the cases, regardless of the interval before surgery.

**DISCUSSION:**

Therapeutic outcomes in our group of elderly patients and/or patients with comorbidities with neoadjuvant short-course RT are such as those of the general population treated with neoadjuvant RT-chemotherapy, all with acceptable toxicity. Therefore, this treatment scheme, with short-course preoperative RT, would be the most appropriate in this group of patients.

## INTRODUCTION

Neoadjuvant radiotherapy (RT) +/− chemotherapy (CT) is the standard treatment of locally advanced rectal cancer because it achieves a greater local control of the disease than surgery alone (1–6).

There are 2 well-established treatment schedules as follows:

1. Radiochemotherapy (RT-CT): RT is administered with a fraction of 1.8–2 Gy in daily sessions to a total dose of 45–50.4 Gy; CT is based on 5-Fluorouracil (5FU), and surgery is scheduled at 6–8 weeks after completing RT-CT.
2. Short-course RT (SCRT): with 5 daily sessions of 5 Gy, with surgery programmed for the next week after finishing RT. The results of the Stockholm III study (7) also allow to establish the interval to surgery of 4–8 weeks.

The interval between the completion of RT-CT and surgery has led to a phenomenon called “tumor regression” (5), which has become crucial in the therapeutic strategy of rectal cancer. Tumor regression is the decrease in the size of the tumor before surgery. During RT, there is damage in tumor DNA and the cell lysis that is derived occurs over the following weeks. Tumor regression may allow surgical resections with free circumferential margin in tumors that, at the time of staging, would have been affected. The response to treatment has also become a major prognostic factor because patients with tumors who respond to neoadjuvant treatment relapse less and survive longer than those who do not respond (5,8,9).

SCRT has good patient compliance and has a better tolerance because of a lower toxicity (1,2,10). Therefore, this regime is especially attractive in frail patients who would hardly tolerate RT-CT. On the other hand, surgery must take place the week after finishing RT. The lack of time interval between RT and surgery did not allow for tumor regression, which would be beneficial in patients with involved mesorrectal fascia (MRF). In this group of patients (involved MRF), and despite the limited evidence published (11,12), it was recommended to delay surgery by 6–8 weeks as in the RT-CT treatment modality. Although waiting for the results of the Stockholm III study (7), in 2012, a randomized Polish study found no difference in survival between the group of patients operated on the week after finishing RT or 4–5 weeks later (13).

In the current study, we present the results of our experience in frail patients treated with short-course RT with and without immediate surgery, regarding the involvement of MRF.

## PATIENTS AND METHODS

In our department, the standard treatment in patients with locally advanced rectal cancer is RT-CT. In 2009, we established a prospective protocol of treatment that included short-course preoperative RT on the primary rectal tumor in frail patients. The inclusion criteria included frail patients defined by the International Society of Geriatric Oncology (see below), mostly older than 80 years of age or with comorbidities that contraindicate CT, with advanced local disease.

Patients diagnosed with an adenocarcinoma of the rectum, with the lower border of the tumor below 15 cm from the anal margin by rigid rectoscopy and/or MRI are staged with thoracic-abdominal CT, endorectal ultrasound, and pelvic MRI. In lower- or middle-rectal tumors in stages II and III, the neoadjuvant treatment (SCRT) is always prescribed. In high-rectal cancer, neoadjuvant treatment is indicated in T4 and/or N2 tumors. These patients are discussed in the multidisciplinary tumor committee, paying special attention to staging and comorbidities.

In patients with uninvolved MRF, surgery was planned within the 2 following weeks after finishing RT. In patients with involved MRF, surgery was delayed and planned 4–8 weeks after finishing RT. In patients operated outside these 2 intervals, the main cause was because of the problems with scheduling surgery.

### Geriatric evaluation

We classified our elderly patients as fit, vulnerable, and frail based on the International Society of Geriatric Oncology to adapt the best treatment to each patient (14,15).

### Radiotherapy

All candidates for neoadjuvant RT underwent a planning CT scan with the patient in a prone position on an open table to allow the displacement of abdominal content outside the irradiation fields. In the clinical volume of planning, we included the rectal tumor with the known nodal involvement, mesorectum, presacral space, and internal and obturator iliac node chains. A 3D conformal technique with a posterior-anterior beam and 2 opposed laterals fields was used to include the planning volume covered by the isodose of 95%. The total dose was 25 Gy in 5 daily fractions prescribed according to the directions of International Commission on Radiation Units 62. Toxicities have been valued according to the criteria of the National Cancer Institute published in the guide Common Terminology Criteria for Adverse Events v 4.0 (http://ctep.cancer.gov).

### Chemotherapy

Patients received CT according to our institution's current guidelines, allowing for the patient's fitness.

Patients with ypT1-2 N0 tumors received fluoropyrimidines (5-fluorouracil or capecitabine) for 6 months. Patients with ypT3-4 and/or N1-2, greater than 70 years received the same adjuvant treatment as ypT1 or ypT2. In patients younger than 70 years, the adjuvant treatment was the FOLFOX regime (5-fluorouracil/leucovorin/oxaliplatin combination) for 6 months.

### Surgery

A total mesorrectal excision (TME) has been the standard surgical option. Anterior resection was the choice when the anal sphincter was not affected. In low-rectal tumors, an anterior ultralow-rectal resection was performed. A diverting ileostomy was indicated for all patients who underwent a rectal anastomosis. In the case of sphincter involvement, an abdominoperineal resection was performed.

### Pathology

A macroscopic description of the surgical specimen and the condition of the MRF was performed. Representative samples of the tumor and tumor-free mucosa were analyzed. The mesorectum was dissected for nodal staging. If no tumor was visible in the surgical specimen but only a residual scar was seen, the whole scar was included in the pathological study. Circumferential resection margin was considered to be involved when the microscopic tumor was less than 1 mm from this margin.

We used the Mandard classification (16) and scored according to a 5-point system for the tumor regression grade after the neoadjuvant treatment.

### Statistics

For clinicopathologic features, *P* values were calculated using the χ^2^ test. Disease-specific survival (DSS) and disease-free survival (DFS) were calculated using the Kaplan-Meier test, and differences between individual curves were evaluated by multivariate analyses using Cox proportional hazards regression models. DSS was defined as the time from surgery until death from cancer (in patients without surgery, the date of diagnosis was the reference date). DFS was defined as the time from radical surgery to the diagnosis of the first recurrence.

## RESULTS

Eighty-seven frail patients diagnosed with rectal cancer were treated in our department with short-course RT between May 25, 2009, and December 31, 2016 (revision date October 4, 2018).

### Patient characteristics are listed

Two patients received neoadjuvant CT sequentially to RT, and 23 patients received postoperative CT (Table 1).

**Table 1.**
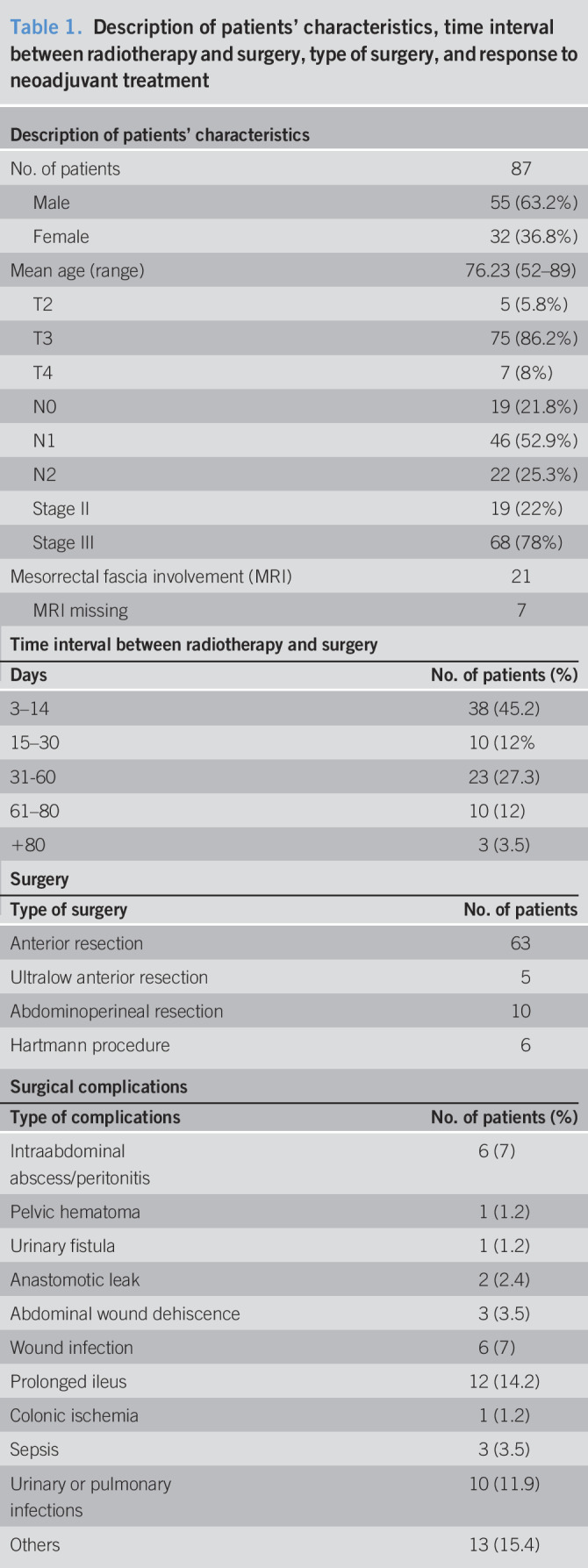
Description of patients' characteristics, time interval between radiotherapy and surgery, type of surgery, and response to neoadjuvant treatment

Acute radiotoxicity was reported in the form of diarrhea in 9 patients and in all cases was grade 2. Regarding late radiotoxicity, 4 patients presented a sacral insufficiency fracture and another 4 patients developed small bowel obstruction (1 grade 3, 2 grade 4, and 1 grade 5). In all, 9% of the patients had late toxicity because of RT with one death.

All patients completed RT, but 3 patients did not proceed to surgery: one patient declined surgery (T3N0, who died from cancer 4.5 years after the end of the RT); one was not fit for surgery; and the third developed systemic disease.

Thirtynine patients (46%) developed surgical complications. Toxicity is described in Table 1. There are no differences in surgical complications based on the intervals to surgery.

Ten patients (11.5%) required reintervention in the immediate postoperative period because of the following reasons: abdominal wound dehiscence (3), anastomotic leak (2), pelvic abscess (2), pelvic hematoma (1), urinary fistula (1), and colonic ischemia (1). Most of these reoperations were carried out on patients who had undergone surgery beyond 30 days after finishing RT (20% on patients who had surgery within 30 days after finishing RT vs 80% when surgery was performed beyond 30 days). There was an immediate postoperative death, and 2 more patients died because of surgical complications at 39 and 41 days after surgery.

We identified 4 cases of positive margins (4.7%). Tumor response grades (TGRs) in our series are referenced in Table 2. No TGR1 had been achieved. TGR2 had only been observed in patients with surgery delayed more than 15 days. TGR4 and TGR5 were more frequent in immediate surgery (61.8% of the 3–30 days' interval). The 2 patients who received neoadjuvant CT had TGR4 and TGR2.

**Table 2.**
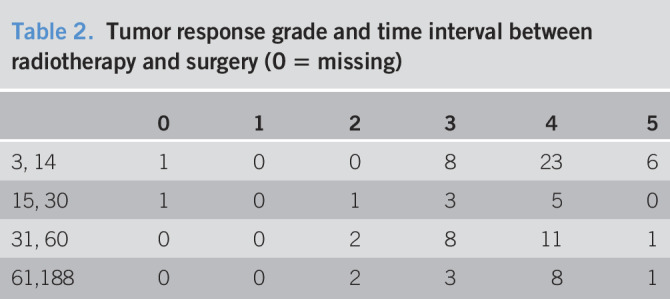
Tumor response grade and time interval between radiotherapy and surgery (0 = missing)

After RT, in the surgical specimens there was a 51% of downstaging and 2.3% of overstaging (Tables 3 and 4). However, 45.4% in patients with clinical stage III had no involved lymph node.

**Table 3.**
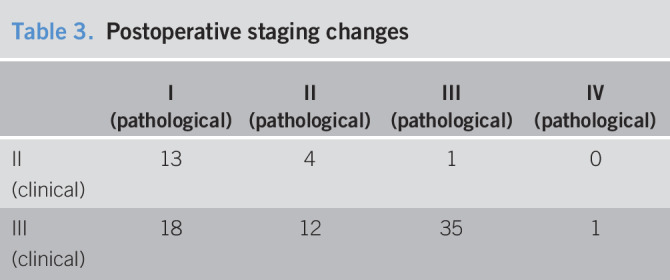
Postoperative staging changes

**Table 4.**
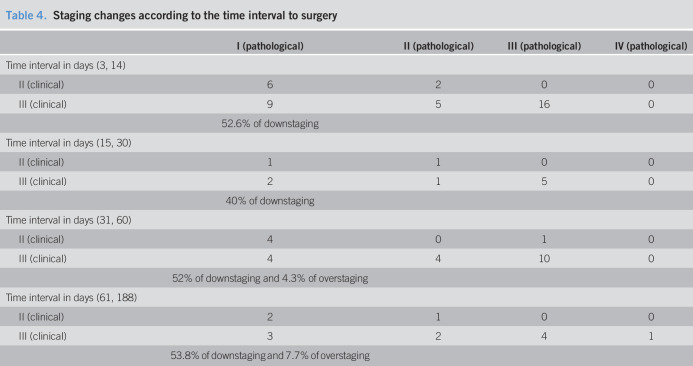
Staging changes according to the time interval to surgery

The mean follow-up was 43.5 months (0.66–106.3), and the median was 36.6 months. At the time of the review, there were 46 patient's disease free (52.8%), 5 patients with disease, 15 patients died from disease progression (17.2%), 1 patient died because of radiotoxicity (1.1%), and 3 patients died in the postoperative period (3.44%). Seventeen patients died from other causes (19.5%).

During the follow-up, 14 patients had a relapse (17.5%), 12 of whom had metastatic disease and the remaining 2 had local recurrence (2.5%).

Two patients with pathological stage I had a distant relapse. No anatomopathological remarkable fact was found in these tumors, except the presence of mucin pools in the surgical specimens. These 2 relapses were very delayed, at 71.3 and 91.13 months from surgery.

**Figure 1.**
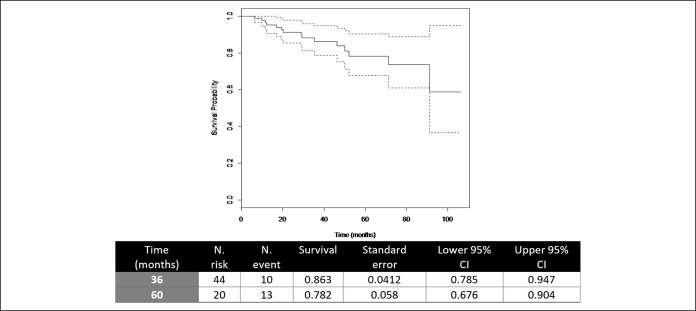
Disease-specific survival. CI, confidence interval.

**Figure 2.**
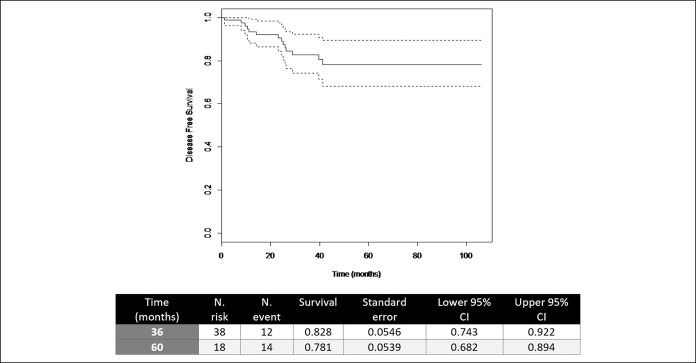
Disease-free survival. CI, confidence interval.

DSS at 36 and 60 months was 86.3% (0.785–0.947) and 78.2% (0.676–0.904), and DFS at 36 and 60 months was 82.8% (0.743–0.922) and 78% (0.682–0.894), respectively (Figures 1 and 2).

## DISCUSSION

The optimum treatment for locally advanced rectal cancer in frail patients because of age or comorbidities is a source of controversies because these patients are not well represented in controlled studies.

Rutten and colleagues (17,18) reviewed the therapeutic results obtained in the Dutch TME study (surgery with TME with or without short-course preoperatory RT) (3) and linked it with data from Dutch Comprehensive Cancer Centers South and West combined. The authors concluded that patients older than 75 years did not benefit from a TME. This has been attributed to the fact that there are higher rates of postoperative deaths at 6 months in older patients than in youngers ones.

On the other hand, there is a systematic review of surgical results in patients treated for rectal cancer (19), and the authors observed that rather than age, the results are limited by the associated morbidity. In older patients with little associated morbidity, complications or mortality rates remain the same as those observed in younger patients. Therefore, a good selection of patients for different therapeutic strategies is of paramount importance at the time of designing the best possible treatment for this population.

Wan et al. (20), based on a Surveillance, Epidemiology, and End Results–registered database, described, in the subpopulation of 75 years old and older, a specific survival at 5 years of 52.1% in patients after surgery alone, 27.7% with RT alone, 70.4% with neoadjuvant RT, and 60.4% with adjuvant RT (*P* < 0.001). They concluded that neoadjuvant RT and surgery are the best treatment modality in older patients, especially in those without serious comorbidities.

Our results confirm the above findings because they do not show any difference from the ones obtained in series with younger patients treated with standard neoadjuvant treatment (RT-CT) and surgery. We emphasize this because in many cases, we choose short-course RT and surgery at the limit of the indication and because of the fact that when we initiated this treatment protocol, the only standard regarding short-course RT was surgery the week after finalizing RT. Because 21 of our patients had an involved MRF, we believed that a longer interval to surgery was needed to allow for tumor regression at a risk of obtaining a positive circumferencial resection margin with the worsening of the therapeutic results that this means (21).

The disease-specific survival of 78% at 5 years with a local recurrence rate of 2.5% in our patients matches those published in a series of younger patients. Therefore, it is possible to make a good selection of patients for this treatment scheme (short-course RT and standard surgery), without detriment to therapeutic results.

Regarding toxicity to RT, we report good acute tolerance to treatment (we only detected G2 diarrheas). By contrast, late toxicity occurred in 9% of cases: 4.5% of incidence of sacral insufficiency fractures and 4.5% of small bowel obstruction with one death. The Stockholm III study (7) described a bowel obstruction rate of 11% and Bujko et al. (10) of 10%. Hatfield et al. (12) also evidenced a death for bowel damage, probably because of RT. The presence of sacral insufficiency fractures as a complication of RT is very well known with a reported 7% incidence. The incidence of its presentation increases with age, in women and a previous history of osteoporosis (22). Becuase our study was carried out, now new RT technologies such as Intensity Modulated Radiotherapy allows to adjust the doses of RT to the tumor volumes better and decreases it at the level of critical organs. This seems to decrease the acute toxicity, and we expect that it will also do so in late toxicity (23).

Regarding postoperative complications, we analyzed the mortality rate beyond 30 days after the recommendations of Rutten and colleagues (17,18). They recommended measuring it at 6 months because, in older patients, death from surgical causes can go beyond 30 days. Our rate is 3.6% (3 patients of 84 who were operated on), which is lower than what they reported (13%). This result agrees with Maas and colleagues who insist that the rates of complications in older patients are more related to comorbidities than to age (24).

We do not find a higher rate of postoperative complications in patients undergoing surgery after a short interval after RT, as described in the Stockholm III study (7). When we separately analyzed the patients who underwent reoperation for surgical complications, we found that they mainly belonged to the group on which surgery was performed after 30 days after the end of RT. A possible explanation for this fact is the bias in our treatment scheme, when selecting patients with involved MRF for delayed surgery. For this reason, in the group of patients with surgery in the short interval (less than 30 days), there are lower T stages than the long interval (we do not find any differences either N stage nor in staging, whether they are II or III). The fact that there are more T2 or favorable T3 in the short interval should not mean protection against surgical complications as Sprenger and colleagues have shown (25). Possibly this finding is more related to the low number of patients in our series rather than another clinical fact with real significance.

In our series, none of the patients achieved a complete remission of the tumor (TGR 1) after RT, and we found a 5.9% of TRG 2 (microscopic residual disease). On the other hand, downstaging occurred in 51% of the cases and in 45.4% of the patients in clinical stage III shifted to pathological stage II. What we want to point out is that downstaging, in our series, occurs at all time intervals to surgery, even in the shortest (3–14 days). It is clear that the longer the interval between the end of RT and surgery, the more likely there is a tumor response (26,27), but tumor responses at short intervals until surgery have been already described; Graf et al. (28) analyzed tumor response in 2 Swedish randomized studies, comparing short-course preoperative RT vs surgery alone or surgery with postoperative RT. Surgery was performed one week after the completion of preoperative RT. The authors found that tumors were smaller, and nodal metastases were less common in the group of patients irradiated before surgery. We have corroborated these findings and demonstrated that downstaging, despite a short interval between RT and surgery, is also feasible. Possibly, the tumors that respond so precociously could be lesions that are highly sensitive to neoadjuvant treatment.

Finally, as mentioned at the beginning of this article, we reaffirm that response to neoadjuvant treatment is a potent prognostic factor and has been repeatedly verified (5,8,9). Despite this fact, we have 2 patients in the current series who have made a good response to treatment in the form of downstaging (in the histological examination, they became stage I), but they relapsed. Nowadays, there is no way to explain this fact, only to point out that those recurrences are late (one case beyond the seventh year after treatment), which shows that the correct follow-up time should include this period.

In stage II-III, our therapeutic results with short-course RT and surgery with TME are similar to those described in series with neoadjuvant RT-CT and standard surgery, despite being elderly patients and/or having comorbidities, all with acceptable toxicity. Therefore, this treatment scheme, with short-course preoperative RT, could be considered the most appropriate in this group of patients.

## CONFLICTS OF INTEREST

**Guarantor of the article:** Maria Cambray.

**Specific author contributions:** Conceptualization, M.C.; methodology, M.C and J.G.; formal analysis, M.C, J.G and M.A.B.; investigation, M.C, M.M, F.L, G.S, R.F and J.C.; writing—original draft preparation, M.C.; writing—review and editing, M.C, J.G, M.A.B, M.M and F.L.; supervision, M.C and F.L; project administration, M.C and E.G. funding acquisition, M.C and F.L.

**Financial support:** This research was funded by Catalan Institute of Oncology.

**Potential competing interests:** None to report.
